# Insight into psychosis in patients in the early stages of psychotic disorders and their parents

**DOI:** 10.1192/j.eurpsy.2025.2141

**Published:** 2025-08-26

**Authors:** L. Gorsic, S. Caratan, H. Gorsic, M. Krevatin, S. Kocijan, V. Grosic, I. Filipcic

**Affiliations:** 1 Day Hospital for non psychotic disorders, University Psychiatric Hospital Sveti Ivan, Zagreb; 2 Department of Psychiatry and Neurology, Faculty of Dental Medicine and Health JJStrosmayer, Osijek; 3 Integrative Psychiatry, University Psychiatric Hospital Sveti Ivan, Zagreb; 4 Department of Psychiatry and Neurologyiy and Neurology, Faculty of Dental Medicine and Health JJStrosmayer, Osijek, Croatia

## Abstract

**Introduction:**

Clinically insight is a multifaceted phenomenon related to acceptance of one’s condition as being psychiatrically ill, recognition of symptoms as pathological, recognition of the social consequences of illness and treatment acceptance. The common features of psychotic disorders are symptoms classified through several dimensions: positive symptoms, negative symptoms, cognitive symptoms, affective symptoms and psychomotor symptoms. These symptoms often cause impaired reality testing and poor insight. Impaired insight is common during early stage of psychosis with its prevalence ranging from around 30-50% and may cause treatment delays, more severe symptoms, poorer treatment adherence, involuntary admissions, potentially aggressive behaviour, poorer social functioning and outcomes. Insight is directly related to the severity of psychotic symptoms and inversely related to depression score. Enviromental factors such as family beliefs and stigma can influence on insight level. Considering that the first symptoms of psychosis often appear in young adulthood while the affected still live with their parents, we believe that the role of parental insight is important in recognizing the signs and treating psychosis.

**Objectives:**

Aim of this study was to investigate level of patient’s and parent’s insight into early psychosis.

**Methods:**

We performed a cross-sectional study on 105 diads parents - children suffering from early stage of the psychotic disorders, during disease < 5 years, diagnoses from the spectrum F20-F29, both sexes, aged 18-35 years who were hospitalized at “Sveti Ivan” Psychiatry Clinic. The purpose and objectives of the research were explained to all participants, and they signed informed consent. The instruments used: sociodemographic questionnaire, The Scale to Assess Unawareness of Mental Disorder - Abbreviated version (SUMD), The Positive and Negative Syndrome scale (PANSS) – The Five factor models, Clinical Global Impression-severity scale (CGI-s). The research was conducted in accordance with Helsinki declaration.

**Results:**

Among the patients, the male gender is slightly more represented, with a good level of education, mostly with the F23 diagnosis, while in the group of parents, mothers are significantly more represented. On the PANSS Five Factor scale, the patients showed significant improvement in all domains. The patients were more aware of their disorder than their parents. The fathers were more aware of their children’s disorders than mothers.

**Conclusions:**

The research has contributed to clarifying the complexity of the issue of insight and complex relationships between parents and children, pointing to the necessity of family psychotherapy and psychosocial interventions in the treatment of patients in the early stages of psychotic disorders.

**Disclosure of Interest:**

None Declared

